# Fiber-Based Scaffolds as Drug Carriers: Recent Advances

**DOI:** 10.3390/pharmaceutics17060740

**Published:** 2025-06-05

**Authors:** Helena P. Felgueiras

**Affiliations:** Centre for Textile Science and Technology (2C2T), University of Minho, Campus de Azurém, 4800-058 Guimarães, Portugal; helena.felgueiras@2c2t.uminho.pt

In recent years, fiber-based materials have been widely explored for their use in biomedical applications, ranging from wound dressings and bone tissue engineering to drug-controlled release and delivery platforms. The increasing prevalence of these materials is attributed to their biocompatibility, biodegradability, and mechanical resilience/resistance, which have made them indispensable in advanced healthcare solutions [1,2]. Among the various biopolymers used, cellulose and its derivatives, along with chitosan and other naturally derived polymers, have played a pivotal role in developing sophisticated drug delivery platforms and tissue engineering scaffolds (Contributions 1 and 2).

The organization and structure of fibers within scaffolds are crucial in determining their efficacy in specific biomedical applications. Different fiber-producing techniques such as electrospinning, wet-spinning, and 3D printing have been explored to optimize these materials for biomedical use (Contributions 1 and 3). Electrospinning, for instance, has emerged as a preferred method for generating fibrous scaffolds that mimic the extracellular matrix (ECM) of human tissues, thereby enhancing cell recognition, adhesion, and proliferation (Contribution 4) [3].

Drug delivery systems have particularly benefited from fiber-based scaffolds. Electrospun nanofibers, with their high surface area and tunable porosity, enable controlled and sustained drug release, reducing systemic side effects and improving therapeutic outcomes (Contribution 2). Studies have demonstrated the efficiency of therapeutic agent-loaded fibrous scaffolds in delivering drugs precisely to target sites, minimizing systemic toxicity and maximizing efficacy (Contribution 1).

Wound healing is one of the key biomedical applications where fiber-based materials have demonstrated significant potential. Due to their ability to promote hemostasis, provide antibacterial protection, and regulate moisture, nanofiber scaffolds are being investigated by many as alternatives to traditional wound dressings (Contribution 2). Moreover, incorporating bioactive agents into these scaffolds has enhanced their healing capabilities, leading to faster tissue regeneration and reduced infection risks. A recent study highlighted the antibacterial and anti-inflammatory properties of electrospun shellac fibers loaded with herbal extracts, showing their potential as advanced wound dressings (Contribution 5) [1]. Additionally, fiber-based scaffolds with embedded nanoparticles, such as zinc oxide (ZnO), have exhibited enhanced antimicrobial activity, making them suitable for treating chronic wounds and diabetic ulcers (Contribution 6). Beyond wound healing, fibrous scaffolds are extensively used in tissue engineering, particularly for tendon, ligament, and bone regeneration. Wet-spun polymeric fibers, for instance, have been identified as potential scaffolds for tendon and ligament repair due to their ability to mimic the native tissue architecture (Contribution 3). Incorporating functional elements such as growth factors has further improved the utility of these scaffolds. For example, scaffolds functionalized with human placental growth factor (PLGF) have demonstrated enhanced osteoplastic material compatibility, paving the way for more effective bone grafting techniques (Contribution 7). Similarly, electrospun polylactic-co-glycolic acid (PLGA) scaffolds modified with antibacterial coatings have shown promising results in preventing infections while promoting cell adhesion (Contribution 4). The development of drug-loaded fibrous scaffolds has revolutionized targeted drug delivery. One of the most promising techniques is hot-melt extrusion, which can be used to embed active pharmaceutical ingredients within multifilament yarns (Contribution 8). This method has enabled the scalable production of fiber-based dosage forms, expanding the potential of fibers in pharmaceutical applications beyond traditional drug delivery systems. Additionally, electrospinning techniques have been refined to facilitate the controlled release of bioactive molecules, including proteins, peptides, and antimicrobial agents. These advances not only enhance drug stability but also allow for the development of patient-specific therapeutic solutions, such as those targeting bacterial activity in diabetic ulcers (Contribution 9).

As research in fiber-based scaffolds continues to evolve, the integration of smart materials, bioactive molecules, and advanced fabrication techniques will drive the next generation of biomedical innovations. The use of biodegradable and biocompatible polymers such as cellulose, chitosan, and PLGA in combination with nanotechnology will further perfect drug delivery mechanisms and tissue engineering applications (Contributions 1 and 2). With ongoing advancements in biomaterials and fiber engineering, fiber-based scaffolds are set to redefine the landscape of regenerative medicine and drug delivery. Future studies should focus on optimizing scaffold design, improving drug-loading efficiencies, and enhancing biocompatibility to maximize their clinical impact. As these materials progress from experimental research to widespread clinical application, they hold promise in significantly improving the outcomes and quality of life of patients [1,2,3].
